# Hepatitis E-related adverse pregnancy outcomes and their prevention by hepatitis E vaccine in a rabbit model

**DOI:** 10.1080/22221751.2019.1643260

**Published:** 2019-07-24

**Authors:** Manyu Li, Shuangshuang Li, Qiyu He, Zhaochao Liang, Lin Wang, Qianhui Wang, Ling Wang

**Affiliations:** a Department of Microbiology and Center of Infectious Disease, School of Basic Medical Sciences, Peking University Health Science Center, Beijing, People’s Republic of China; b Department of Infectious Diseases, Taiyuan No. 3 Hospital, Taiyuan, Shanxi Province, People’s Republic of China

**Keywords:** Hepatitis E, pregnancy, HEV vaccine, adverse pregnancy outcomes, vertical transmission

## Abstract

Hepatitis E virus (HEV) can lead to high mortality during pregnancy. This study was to investigate the adverse pregnancy outcomes caused by different HEV genotypes and their prevention by HEV 239 vaccine in rabbits. Forty-two female rabbits were randomly and equally divided into 7 groups (A-G). HEV 239 vaccine and a placebo were administered to groups E (10 μg×2), F (5 μg×2) and G (1 mL of PBS×2) before copulation. After pregnancy, 1 mL of 1.5×106 copies/mL rabbit HEV3 was inoculated to groups A, E, F and G, swine HEV4/human HEV3 to groups B/C, and group D was a negative control. Anti-HEV antibody, HEV RNA, and alanine aminotransferase (ALT)/aspartate aminotransferase (AST) levels were monitored. Pregnant rabbits infected by HEV manifested HEV infection symptoms including fecal virus shedding, ALT/AST elevation, and histopathological changes, and adverse pregnancy outcomes. Immunized pregnant rabbits in groups E and F showed no HEV infection symptoms and adverse outcomes. The newborn rabbits delivered by pregnant rabbits with/without immunization showed without/with HEV infection symptoms. This study demonstrated that multiple genotypes of HEV infection can cause adverse outcomes and HEV 239 vaccine can prevent HEV-related adverse outcomes in pregnant rabbits.

## Introduction

Hepatitis E virus (HEV) is the main cause of acute viral hepatitis in developing countries, which has aroused wide public concern [1–3]. Each year, there are approximately 20 million people infected with HEV worldwide, causing 70,000 deaths [4]. HEV, a single-stranded positive RNA virus, spreads through mainly the fecal-oral route and has been recognized as a zoonotic pathogen [5]. HEV can be divided into 8 genotypes (HEV1-8), among which HEV1-4 are closely related to human infection. HEV1 and HEV2 can only infect humans, while HEV3 and HEV4 are zoonotic [1–3].

HEV often causes acute self-limited disease, with a mortality of approximately 0.2–1% [3]. However, in pregnant women, the mortality rate of HEV infection can reach as high as 25% [6–9]. HEV infection in pregnant women can also lead to adverse pregnancy outcomes including miscarriage, stillbirths, fulminant hepatic failure and membrane rupture [10]. However, to date, the mechanism of the high mortality rate of HEV infection in pregnant women remains unclear. HEV-related adverse pregnancy outcomes are caused by mainly HEV1 [10], and whether other genotypes of HEV can lead to adverse outcomes during pregnancy is still unclear. In addition to HEV genotypes, the pathogenesis of HEV-related adverse pregnancy outcomes may be related to changes in cellular immunity and hormones during pregnancy [10], but that possibility warrants further investigation. Currently, there is no recommended drug for HEV-infected pregnant women therefore, an effective prevention strategy is of high priority for this population. Although the HEV 239 vaccine (Hecolin®), which is a bacterially expressed recombinant peptide corresponding to amino acid residues 368–606 of ORF2 of HEV1, has been commercialized in China and can successfully prevent HEV infection for years [11], it still lacks studies on its efficacy for adverse pregnancy outcomes. Previously, we successfully established a HEV-infected pregnant rabbit model that can simulate the HEV-related high mortality rate observed in pregnant women [12]. Therefore, this study aimed to investigate the pathogenicity of different HEV genotypes in pregnant rabbits and the efficacy of the HEV 239 vaccine in preventing adverse outcomes and vertical transmission of HEV infection in pregnant rabbits.

## Materials and methods

### Ethics statement

The animal experiments were approved by the Committee of Laboratory Animal Welfare and Ethics, Peking University Health Science Center, and were in strict accordance with the protocol for the review of the Laboratory Animal Welfare and Ethics, Peking University Health Science Center (IACUC number: LA2017047).

### Animals

Forty-two 7-month-old female Japanese white rabbits weighing between 5.0 and 6.0 kg were obtained from the Department of Laboratory Animal Science of Peking University Health Science Center. Serum and feces specimens from selected rabbits were collected once a week for 2 weeks to test ALT and AST levels and to establish a baseline. Enzyme-linked immunosorbent assay (ELISA) and reverse transcription-nested PCR (RT-nPCR) were performed to ensure that all specimens were negative for HEV RNA and antibodies against HEV.

### Vaccines

The HEV 239 vaccine (Hecolin; Xiamen Innovax Biotech, Xiamen, China) is a 26 kDa recombinant polypeptide expressed by the *Escherichia coli system* derived from the 368–606 aa segment of the HEV1 (GenBank D11092) ORF2 protein. It is currently the only commercially available HEV vaccine globally. To date, the HEV 239 vaccine is only available in the Chinese market and has been approved for use in people older than 16 years, which is indicated for vaccinating individuals at high risk of HEV infection [11].

### Viruses

The rabbit HEV strain (CHN-BJ-R14, genotype 3, GenBank JQ768461), swine HEV strain (CHN-SD-SW2, genotype 4, GenBank KP284140) and human HEV strain (CHN-SH-W, genotype 3, GenBank MF996356) used in this study were all isolated from feces. Inocula were prepared as described previously [12]. The viral load of each HEV strain was adjusted to 1.5×10^6^ copies/mL by using a one-step real-time quantitative PCR assay [12]

### Experimental design

All selected female rabbits (*n* = 42) were randomly divided into 7 groups (A-G), with 6 rabbits per group (Supplementary Fig. 1). Before copulation, rabbits in groups E and F were inoculated intramuscularly with a 10 and 5 μg dose of HEV 239 vaccine on weeks 0 and 4 respectively, while rabbits in group G as a vaccine control group were inoculated with a sterile PBS solution. On week 7, all female rabbits (*n* = 42) copulated with healthy male rabbits. After their pregnancy was confirmed [12], rabbits in groups A, E, F, and G were inoculated intravenously with 1 mL of 1.5×10^6^ copies/mL rabbit HEV strain (rHEV3). Rabbits in groups B and C were inoculated intravenously with 1 mL of 1.5×10^6^ copies/mL swine HEV4 strain (sHEV4) and human HEV3 strain (hHEV3), respectively, and rabbits in group D were inoculated intravenously with a rabbit feces suspension without HEV infection as a negative control.

### Sample collection and processing

After virus inoculation, serum and fecal specimens from pregnant rabbits were collected weekly for serum ALT and AST level, fecal HEV RNA load, and serum estrogen and progesterone level testing. Three weeks after newborn rabbits were born, their serum and fecal samples were collected weekly for serum ALT and AST level and fecal HEV RNA load testing. Livers, ovaries, or placentas were collected immediately from dead HEV-infected pregnant rabbits, euthanized HEV-infected pregnant rabbits, dead newborn rabbits and euthanized HEV-infected young rabbits. Each tissue was stored at −80°C for detection of HEV RNA positive and negative strands and HEV viral load. In addition, the tissue specimens were also processed for histopathology, immunofluorescence and immunohistochemistry (IHC) by being immediately fixed in 10% neutral buffered formalin [12].

### Detection of pregnancy-related hormones

To confirm the female rabbits’ pregnancy, their serum (day 0, day 10, day 17 and day 24 after pregnancy) and postpartum serum were all tested for progesterone and estrogen by the chemiluminescent enzyme immunoassay method on a Siemens Immulite 2000 Immunoassay System according to the manufacturer’s instructions [12].

### Detection of ALT and AST concentrations

All serum samples were tested immediately for ALT and AST concentrations using standard methods on a Hitachi Automatic Clinical Analyzer 7180. When the ALT or AST level became more than 2 times the preinfection baseline value, the rabbit was considered to have liver inflammation [13].

### Evaluation of anti-HEV antibodies by ELISA and quantitative detection

All rabbit serum samples were detected by ELISA for total anti-HEV antibodies according to the manufacturer’s instructions (Wantai, Biopharmaceutical, Beijing, China). When the S/CO value > 1, it was considered to be positive [14]. The geometric mean antibody titre (GMT) and quantitation of anti-HEV antibodies were performed as previously described [15].

### Extraction and detection of HEV RNA

Total RNA in serum and fecal suspensions was extracted according to the RNAgents Total RNA Isolation System Kit (Promega, Madison, USA) instructions, and HEV RNA fragments were amplified by RT-nPCR [16]. RT-nPCR was carried out to detect HEV RNA as previously described [16]. Tissues with detectable positive-stranded HEV RNA were then assayed for negative-sense HEV RNA by RT-nPCR with the same 2 sets of universal primers, which used the external forward primer for cDNA synthesis and the amplification conditions were the same as those used in the detection of positive-sense HEV RNA [16].

### Real-time fluorescence quantitative PCR

According to the literature [17], real-time fluorescence quantitative PCR (RT-qPCR) in this study was performed by using the Taqman probe detection method and QuantiTect® Probe RT–PCR Kit (Qiagen, Hilden, Germany).

### Histopathology, immunofluorescence and immunohistochemistry staining

For histopathology, tissue samples were prepared for hematoxylin and eosin (HE) staining, IHC (microscope equipped with a digital camera, Olympus CX31, Japan) and immunofluorescence (Nikon DS-U3, Japan) by first being fixed in 10% neutral buffered formalin immediately following sampling according to the literature [18,19]. HEV proteins were visualized by using HEV ORF2- and ORF3-specific antibodies (1:1600, Bioss, Woburn, USA). Nuclear staining was achieved with DAPI (Beyotime, Shanghai, China). CD4^+^ and CD8^+^ cells were visualized with anti-CD4^+^ (1:1000, Bio-Rad, California, USA) and -CD8^+^ polyclonal antibodies (1:1500, Goodbio Technology, Wuhan, China).

### Statistical analysis

Statistical analysis was performed using the SPSS PASW Statistics v18.0 statistical software package (SPSS, Inc., USA, http://www.ibm.com/cn/). Data were compared using Student’s t-test or chi-squared test. A *P*-value of <0.05 was considered significant.

## Results

### Pathogenicity of HEVs infection in pregnant rabbits

To analyze and compare the results of different genotypes of HEV (HEVs) infection in pregnant rabbits, we inoculated pregnant rabbits in groups A, B and C with rabbit HEV3 (rHEV3), swine HEV4 (sHEV4), and human HEV3 (hHEV3) respectively after the pregnancy was confirmed. Group D was set up as a negative control group without HEV infection.

Different incidences of adverse pregnancy outcomes were observed among groups A-D. The overall incidence of adverse pregnancy outcomes in group A (rHEV3) was 83.3% (5/6), with 16.7% (1/6) maternal death, 50% (3/6) stillbirth, and 16.7% (1/6) miscarriage; in group B (sHEV4) was 83.3% (5/6), with 66.7% (4/6) stillbirth and 16.7% (1/6) miscarriage; and in group C (hHEV3) was 66.7% (4/6), with 16.7% (1/6) maternal death and 50% (3/6) stillbirth. All rabbits in group D had normal delivery and no adverse pregnancy outcomes occurred. While maternal death only occurred in groups A (16.7%) and C (16.7%) and miscarriage was only observed in groups A (16.7%) and B (16.7%), stillbirth was observed in groups A (50%, 32.3 ± 1.5 days post pregnancy), B (66.7%, 31.5 ± 1.3 days post pregnancy) and C (50%, 30.5 ± 1.3 days post pregnancy). All HEV genotypes could induce adverse pregnancy outcomes in pregnant rabbits, and there was no statistically significant difference in the incidence of adverse outcomes among groups A, B, and C (*P* > 0.05) (Table 1).

**Table 1.** Adverse pregnancy outcomes of pregnant rabbits infected by different genotypes of HEV.

Groups (NO., HEV genotypes)	Adverse pregnancy outcomes^a^ (%)			Normal delivery^b^ (*n*^c^, %)	*P* value^d^
	Death	Stillbirth	Miscarriage		
A (6, rHEV3)	1/6^e^(16.7)	3/6(50)	1/6(16.7)	1/6(4, 16.7)	0.015*
B (6, sHEV4)	0/6(0)	4/6(66.7)	1/6(16.7)	1/6(5, 16.7)	0.015*
C (6, hHEV3)	1/6^e^(16.7)	3/6(50)	0/6(0)	2/6(7, 33.3)	0.060
D (6, placebo)	0/6(0)	0/6(0)	0/6(0)	6/6(38, 100)	–
E (6, rHEV3)	0/6(0)	0/6(0)	0/6(0)	6/6(40, 100)	1.000
F (6, rHEV3)	0/6(0)	1/6(16.7)	0/6(0)	5/6(35, 83.3)	1.000
G (6, rHEV3)	1/6(16.7)	3/6(50)	2/6(33.3)	0/6(0, 0)	0.002*

^a^Number of rabbits with adverse pregnancy outcomes/total number of rabbits in the group.

^b^Number of rabbits had normal delivery/total number of rabbits in the group.

^c^Number of newborn rabbits in the group.

^d^Compared with group D.

^e^The rabbits in group A and C died 34th day and 31st day post pregnancy.

*Significantly different, *P* < 0.05.

**Figure 1.**
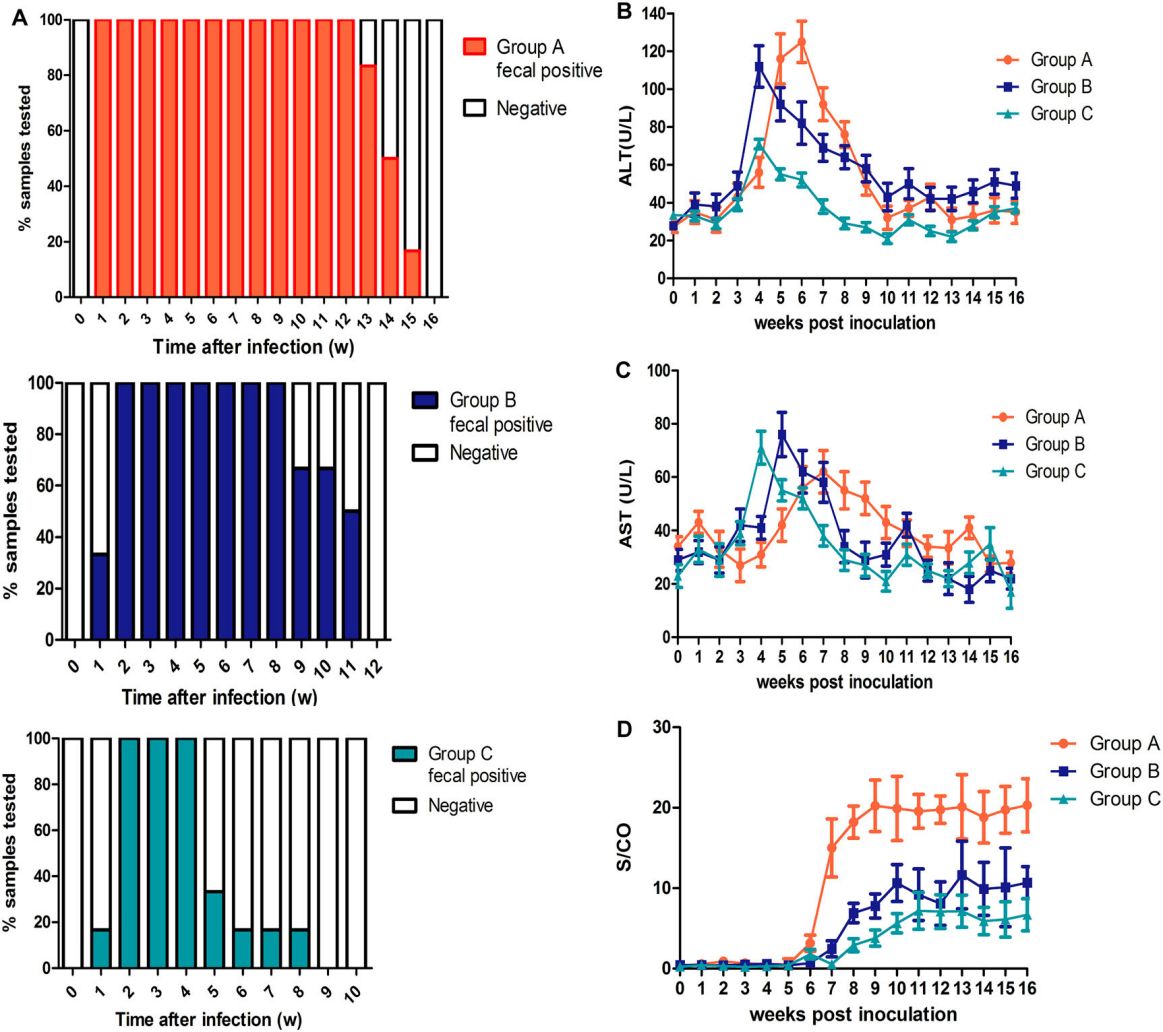
**Duration of fecal virus shedding and dynamic seroconversion of anti-HEV antibodies, ALT and AST of pregnant rabbits**. Duration of fecal HEV RNA shedding among group A, B and C. (B) Dynamic ALT changes among three groups. (C) Dynamic AST changes among three groups. (D) Dynamic seroconversion of anti-HEV antibodies among three groups.

Different durations of fecal virus shedding were observed in groups A-C. Rabbits in group A (rHEV3) began fecal virus shedding at 1 week post inoculation (wpi) that lasted for an average of 13.5 ± 0.4 weeks; rabbits in group B (sHEV4) began shedding at 2wpi that lasted for 10.2 ± 0.5 weeks on average; and rabbits in group C (hHEV3) began shedding at 2wpi that lasted for 4.8 ± 0.7 weeks on average. The durations of fecal virus shedding were statistically different among the three groups, with group A shedding (13.5 ± 0.4 w) being significantly longer than that of group B (10.2 ± 0.5 w) and group C (4.8 ± 0.7 w) (*P* < 0.05) (Table 2 and Figure 1). The sequences of HEV detected in group A, B and C feces were all in accordance with the inoculation viruses.

**Table 2.** Markers of HEV infection in pregnant rabbits.

Groups (NO., HEV genotypes)	Mean duration of fecal virus shedding ± SD (week)	No. of positive rabbits/total no. of rabbits tested post-inoculation						
		Shedding virus	Viremia	+/- stranded HEV RNA in liver^a^	+/- stranded HEV RNA in placenta^b^	Anti- HEV (+)	ALT level (peak/pre ≥2)	AST level (peak/pre ≥2)
A (6, rHEV3)	13.5 ± 0.4*	6/6	1/6	6/6	3/4^d^	6/6	4/6	3/6
B (6, sHEV4)	10.2 ± 0.5	6/6	0/6	6/6	0/5^d^	6/6	3/6	1/6
C (6, hHEV3)	4.8 ± 0.7	6/6	0/6	3/6^d^	0/5^d^	4/6	1/6	0/6
D (6, placebo)	–	0/6	0/6	0/6	0/6	0/6	0/6	0/6
E (6, rHEV3)	–	0/6	0/6	0/6	0/6	6/6^c^	0/6	0/6
F (6, rHEV3)	–	0/6	0/6	0/6	0/6	6/6^c^	0/6	0/6
G (6, rHEV3)	12.0 ± 1.4	6/6	1/6	6/6	1/3	6/6	3/6	3/6

^a^Positive and negative stranded HEV RNA in liver were both detected.

^b^Positive and negative stranded HEV RNA in placenta were both detected.

^c^After HEV 239 vaccination and before virus inoculation.

^d^Only positive stranded HEV RNA were detected in 3/6 of livers in group C, 1/4 of placentas in group A, 2/5 of placentas in group B, 2/5 of placentas in group C.

*Group A was significantly higher than that of group B and group C.

In group A, 16.7% (1/6) of pregnant rabbits had viremia, while the rabbits in the other groups did not. Positive and negative HEV RNA strands in the livers and placentas were detected in 100% (6/6) and 50% (3/6) of group A’s rabbits respectively; positive and negative RNA strands were detected in the livers of 100% (6/6) and 50% (3/6) of the rabbits in groups B and C respectively. In groups A, B and C, 66.7% (4/6), 50% (3/6) and 16.7% (1/6) of rabbits showed a significant increase in ALT levels that was more than 2 times the baseline value respectively, while AST levels of 50% (3/6), 16.7% (1/6) and 0% (0/6) of rabbits in groups A, B and C significantly increased more than 2 times the baseline value respectively. In addition, seroconversion of anti-HEV antibodies in groups A (6/6), B (6/6), and C (4/6) was observed, and S/CO values in group A (20.3 ± 3.3) were significantly higher than those in groups B (10.7 ± 2.1) and C (6.1 ± 1.9) (*P* < 0.05) (Table 2 and Figure 1).

### Characteristics of histopathology and immunofluorescence of HEVs infection

**Figure 2.**
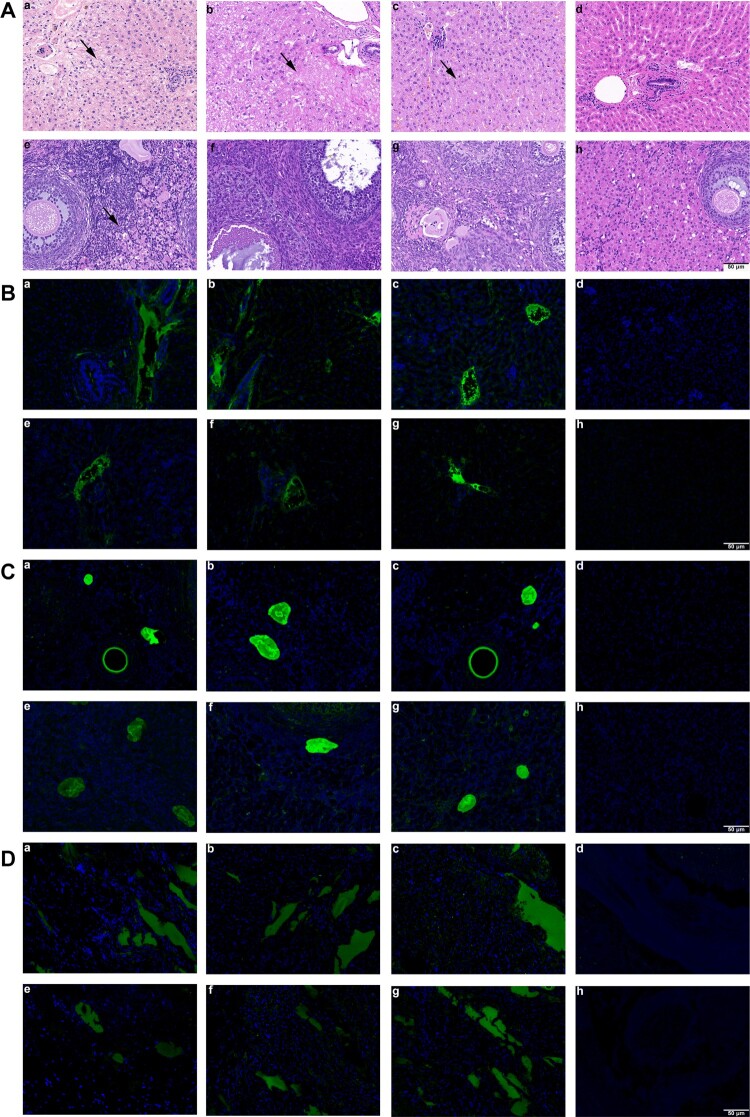
**Histopathology and immunofluorescence of pregnant rabbits in group A, B, C and D**. Liver and ovary histopathology. (a-c) HE staining showed hepatocyte degeneration and necrosis, collagen fibre hyperplasia and fibrosis, and infiltration of inflammatory cells in liver sections of group D, E and F rabbits; (d) liver section of group G showed no visible pathological changes; (e) mild inflammatory cells’ inflitration in ovary section of group A; (f-h) no visible pathological signs of HEV infection in ovary sections of group B-D rabbits. (B) Immunofluorescence staining of HEV ORF2 and ORF3 in liver. (a-c, e-g) Positive signals of HEV ORF2 and ORF3 respectively in liver sections of rabbits in group A-C; (d, h) no positive signals of HEV ORF2 and ORF3 were observed in liver section of rabbits in group D. (C) Immunofluorescence staining of HEV ORF2 and ORF3 in ovary. (a-c, e-g) Positive signals of HEV ORF2 and ORF3 respectively in ovary sections of rabbits in group A-C; (d, h) no positive signals of HEV ORF2 and ORF3 were observed in ovary section of rabbits in group D. (D) Immunofluorescence staining of HEV ORF2 and ORF3 in placenta. (a-c, e-g) Positive signals of HEV ORF2 and ORF3 respectively in placenta sections of rabbits in group A-C; (d, h) no positive signals of HEV ORF2 and ORF3 were observed in placenta section of rabbits in group D. Original magnification,×200.

To clarify the histopathological changes in pregnant rabbits’ tissues, we performed HE staining on rabbit livers and other organs (Figure 2). In group A, hepatic hemorrhage was found in livers of pregnant rabbits, with a large number of red blood cells in hepatic sinusoids, extensive edema, degeneration and necrosis of hepatocytes and inflammatory cell infiltration; necrosis and degeneration of hepatocytes with inflammatory cell infiltration in liver tissue of pregnant rabbits of groups B and C were also observed, but the degree of inflammation was milder than that of group A; and no obvious pathological change in the liver tissue was observed in group D. In addition, the ovary tissue of group A showed slight inflammatory cell infiltration, but groups B, C and D showed no obvious pathological changes in ovary tissues.

To assess the replication efficiency of different genotypes of HEV in the liver, ovary and placenta, the expression of HEV ORF2 and ORF3 was detected by immunofluorescence staining. HEV ORF2 and ORF3 antigens were positive in the liver, ovary, and placenta of rabbits in groups A, B, and C but not in the organs of group D (Figure 2).

### The estrogen levels and cellular immunity in HEV-infected pregnant rabbits

By regression analysis of serum estrogen levels and fecal viral load in group A after virus inoculation, a positive correlation between serum estradiol levels and fecal viral load in pregnant rabbits (*r*^2 ^= 0.4339) was observed, which indicated that estrogen levels were correlated with HEV infection and the higher the estrogen level, the higher the fecal viral load in pregnant rabbits was (Supplementary Fig. 2A).

To investigate the relationship between HEV infection and cellular immunity in pregnant rabbits, we investigated the cellular immune response associated with HEV infection in the liver, ovary, and placenta through IHC. IHC experiments on CD4^+^ and CD8^+^ T cells showed that the signals of CD4^+^ T cells and CD8^+^ T cells in the liver, ovary and placenta of pregnant rabbits infected with rHEV3 were all positive, and the expression of CD8^+^ T cells was higher than that of CD4^+^T cells, but pregnant rabbits of group D showed low but similar levels of CD4+ T cells and CD8+ T cells in liver, ovary and placenta (Supplementary Fig. 2B).

### HEV infection in newborn rabbits and vertical transmission

By analyzing HEV infection in surviving newborn rabbits delivered by pregnant rabbits infected with different genotypes of HEV, 50% (2/4) of surviving newborn rabbits of group A had fecal virus shedding after birth, 25% (1/4) were positive for anti-HEV antibody without HEV infection and 25% (1/4) did not have HEV infection; in surviving newborn rabbits of group B, 20% (1/5) had viremia after birth and 80% (4/5) did not have HEV infection; and in group C’s surviving newborn rabbits, 100% (7/7) were without HEV infection. The HEV infection rate of surviving newborn rabbits in group A was significantly higher than that of surviving newborn rabbits in group C (*P* < 0.05) (Supplementary Table 1).

**Figure 3.**
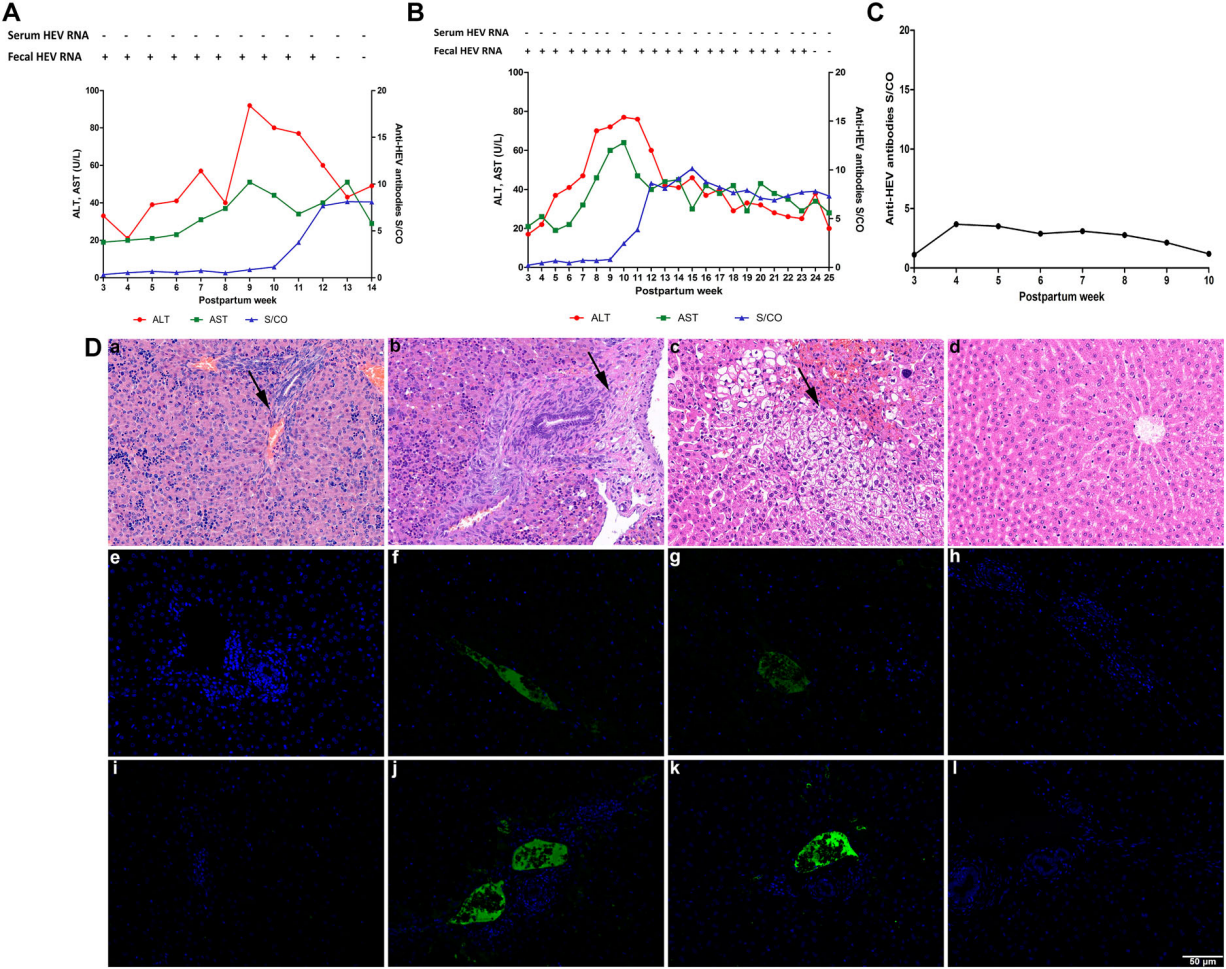
**Dynamic seroconversion of anti-HEV, HEV RNA, ALT, AST, histopathology and immunofluorescence of newborn rabbits.** and (B) Dynamic seroconversion of antiHEV, HEV RNA, ALT and AST of group A newborn rabbits Aa and Ab with fecal HEV RNA shedding. (C) Dynamic seroconversion of anti-HEV of group A newborn rabbits Ac with anti-HEV antibodies. (D) Liver histopathology and Immunofluorescence staining of HEV ORF2 and ORF3 in liver of newborn rabbits. (a, d) Liver section of group A newborn dead rabbit and group D newborn rabbit showed no visible pathological signs of HEV infection; (b, c) various pathological changes in liver sections of group A newborn rabbits Aa and Ab respectively; (e, i, h, l) negative signals for HEV ORF2 and ORF3 antigen in liver of group A newborn dead rabbit and group D newborn rabbit; (f, g, j, k) positive signals for HEV ORF2 and ORF3 antigen in livers from group A newborn rabbits Aa and Ab. Original magnification,×200.

One newborn rabbit of group A (Aa) shed virus in its feces for 12 weeks, and the other (Ab) shed for 23 weeks. Both ALT and AST levels of these two rabbits significantly increased more than 2 times the baseline value and seroconversion of anti-HEV antibodies was also observed (Figure 3A and 3B). The third newborn rabbit (Ac) maintained a low level of anti-HEV antibodies that decreased over time (Figure 3C).

The liver tissues of euthanized newborn rabbits were analyzed by histopathology and immunofluorescence. Structural disorder, hepatocyte necrosis and inflammatory cell infiltration were observed in the liver tissue of Aa and extensive hepatocyte edema, balloon-like degeneration and hepatocyte necrosis were observed in the liver tissue of Ab. Immunofluorescence analysis showed positive signals for HEV ORF2 and ORF3 in the liver tissues of Aa and Ab (Figure 3D).

### Antibody responses after vaccination

Different doses of the HEV 239 vaccine were administered to pregnant rabbits to evaluate the protective efficacy. Four weeks after the first immunization in groups E and F, the serum anti-HEV antibody-positive rates of rabbits inoculated with the 10 and 5 μg dose HEV 239 vaccine were 83.3% and 66.7%, respectively. After the whole immunization process, all vaccinated rabbits were positive for anti-HEV antibodies, whereas the rabbits of the placebo group, G, were all negative for anti-HEV antibodies (Table 3).

**Table 3.** The seroconversion rates of anti-HEV antibody after vaccination.

Groups (No.)	HEV 239 (μg)	The seroconversion rates of anti-HEV antibody (%)		
		1 week^a^	4 week^b^	5 week^c^
E (6)	2×10	2/6(33.3)	5/6(83.3)	6/6(100)
F (6)	2×5	1/6(16.7)	4/6(66.7)	6/6(100)
G (6)	2×0	0/6(0)	0/6(0)	0/6(0)

^a^A week after the first immunization.

^b^Before the second immunization.

^c^A week after the second immunization.

**Figure 4.**
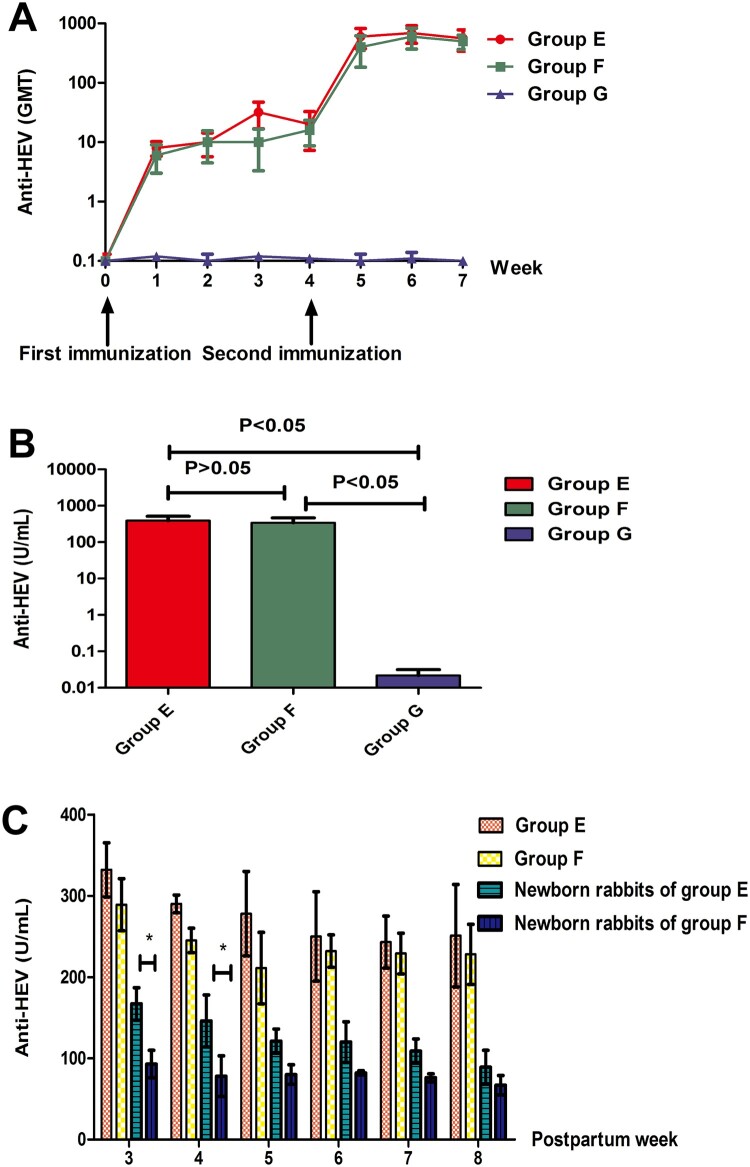
**Antibody responses of rabbits after vaccination.** (A) Dynamics of GMT induced by various types of vaccination. (B) The comparison of anti-HEV levels in each group. Y axis represents anti-HEV levels on week 7 expressed in WHO unit/ml. (C) Postpartum protection of rabbits and newborn rabbits. Y axis represents anti-HEV levels on postpartum week 3 to week 8 expressed in WHO unit/ml (* represents *P* < 0.05).

To evaluate the dynamics of anti-HEV antibodies induced by different doses of the HEV 239 vaccine throughout the whole immunization process, the GMT was measured weekly. The GMT induced by the 10 and 5 μg dose vaccines gradually increased after the first immunization and increased rapidly after the second immunization (Figure 4A).

Three weeks after the whole immunization process, the anti-HEV antibody concentrations of rabbits’ serum were quantified and the average anti-HEV antibody concentrations were compared among the groups. The results showed that the average anti-HEV antibody concentrations between group E and group F were not significantly different (*P* > 0.05) and that the average anti-HEV antibody concentrations in groups E and F were higher than that in group G (*P* < 0.05) (Figure 4B).

### Protective efficacy of the HEV 239 vaccine in pregnant rabbits

After pregnancy was confirmed, rabbits in groups E, F and G were inoculated with the rHEV3 virus on the 10th day post copulation. None of the rabbits in groups E and F developed HEV infection symptoms, such as viremia, fecal virus shedding and elevation of ALT and AST levels, with only one rabbit in group F having a stillbirth. In contrast, all rabbits in group G began seroconversion to anti-HEV antibodies and fecal virus shedding at 2 wpi. Adverse pregnancy outcomes, including miscarriage, stillbirth, and death, occurred at 4 wpi, indicating that HEV can successfully infect rabbits without HEV 239 vaccination.

Statistical analysis showed that compared with placebo administration, two doses of the 10 μg HEV 239 vaccine not only protected pregnant rabbits from rabbit HEV infection but also prevented the occurrence of adverse pregnancy outcomes (*P* < 0.05). Two doses of the 5 μg HEV 239 vaccines could protect pregnant rabbits from rabbit HEV infection, with only one case of stillbirth occurring, which also had a significant difference compared with placebo administration (*P* < 0.05) (Table 4).

**Table 4.** Protection of rabbits against challenge with rHEV-3.

Groups (No.)	HEV 239 (μg)	Pre-challenge	Post challenge			
		Mean anti-HEV level ± SD(U/mL)	Number of rabbits shedding virus in feces^a^	Ab resp.^b^	Number of rabbits with adverse pregnancy outcomes^c^	*P* values^f^
E (6)	2×10	389.2 ± 112.3	0/6	−	0/6	0.002*
F (6)	2×5	339.5 ± 165.2	0/6	−	1/6^d^	0.015*
G (6)	2×0	<1	6/6	+ (>4fold)	6/6^e^	−

^a^Number of rabbits shedding virus in feces/total number of rabbits in the group.

^b^Ab resp. (+) represents the mean anti-HEV level in every group post challenge is four times higher than that pre-challenge.

^c^Number of rabbits with adverse pregnancy outcomes/total number of rabbits in the group.

^d^One rabbit had stillbirth.

^e^One rabbit died, three rabbits had stillbirth and two rabbits had miscarriage.

^f^The vaccine efficacy compared to group G.

*Significantly different, *P* < 0.05.

Serum anti-HEV antibody levels of newborn rabbits in groups E and F were followed. From the 3rd to 8th week after birth, the levels of anti-HEV antibodies continued to decrease and then remained at the same level, while the anti-HEV antibody levels in the pregnant rabbits of groups E and F decreased slightly and remained at the same level. The initial level of anti-HEV antibody in newborn rabbits in group E was higher than that in group F but remained at the same level eventually (Figure 4C).

## Discussion

Although studies have suggested that mainly HEV1 infection is associated with high mortality in pregnant women [10], there is limited research to investigate the influence of other HEV genotypes. In this study, the incidences of adverse pregnancy outcomes were not significantly different among the three groups of rabbits infected with rHEV3, sHEV4 and hHEV3, indicating that different genotype HEVs with the same viral load can all cause adverse pregnancy outcomes. This study is the first time that rabbits have been able to be infected by hHEV3, which may be due to the use of different subgenotypes compared with other studies [20] or because that pregnant rabbits are more prone than nonpregnant rabbits to hHEV3 infection. However, the manifestations of different HEV genotypes infection, including fecal virus shedding duration, the anti-HEV S/CO levels and pathological changes, were different among the groups, and rabbits infected with rHEV3 showed more obvious symptoms than the other groups.

The vertical transmission of HEV has always attracted much attention [21–23], and several studies have shown that HEV can replicate in the human placenta [11,24]. In this study, the positive and negative strands of HEV RNA were detected in placentas and immunofluorescence results showed that both HEV ORF2 and ORF3 signals were positive in the placenta, suggesting that HEV may replicate in the placenta, possibly leading to vertical transmission. We also found that the ovaries of HEV-infected pregnant rabbits were positive for HEV ORF2 and ORF3, indicating that HEV may also replicate in rabbit ovaries. Two of the surviving newborn rabbits (Aa and Ab) delivered by HEV-infected pregnant rabbits showed symptoms of HEV infection, suggesting possible vertical transmission.

The alteration in hormone secretion during pregnancy may be associated with hepatitis E-related poor pregnancy outcomes, and estrogen has been proven to be able to promote HEV replication in vitro [25]. Our results showed that the estrogen level was positively correlated with fecal viral load, suggesting that estrogen may be able to promote HEV replication in pregnant rabbits. The decrease in the CD4/CD8 ratio in blood may be related to high mortality and adverse outcomes during pregnancy caused by HEV infection [26,27]. In this study, in accordance with the lowered CD4/CD8 cell ratio in blood samples [26,27], the IHC results of tissues showed that compared with the negative control rabbits, pregnant rabbits infected with HEV were positive for both CD4^+^ and CD8^+^ T cells in the liver, ovary, and placenta and that the proportion of CD8^+^ T cells was higher than that of CD4^+^ T cells.

In this study, similar to previous studies [28,29], our results showed that the HEV 239 vaccine can effectively prevent rabbit HEV infection in pregnant rabbits. For the first time, our results showed that the HEV 239 vaccine can also prevent the occurrence of adverse pregnancy outcomes in pregnant rabbits. Different doses of vaccination in pregnant rabbits could produce anti-HEV antibodies without significant differences in immunization efficacy. We also noted that anti-HEV antibodies could be detected in newborn rabbits delivered by immunized pregnant rabbits, which may be related to breastfeeding of female rabbits. The anti-HEV antibody persisted in newborn rabbits, and different doses of the HEV 239 vaccine had the same efficacy on newborn rabbits.

In summary, our results showed that different genotypes of HEV can infect pregnant rabbits and lead to adverse pregnancy outcomes and vertical transmission. The HEV 239 vaccine can effectively protect pregnant rabbits from rabbit HEV infection and adverse pregnancy outcomes. Although the animal numbers in the present study are limited, our results still suggest that to avoid hepatitis E-related adverse pregnancy outcomes and vertical transmission, childbearing-age women should be immunized with the HEV vaccine before pregnancy.

## Supplementary Material

Supplemental Material
