# Supplementary material for: Hepatitis E-related adverse pregnancy outcomes and their prevention by hepatitis E vaccine in a rabbit model
Source: Emerg Microbes Infect. 2019 Jul 24;8(1):1066–75. doi: 10.1080/22221751.2019.1643260 (PMC6711181; doi:10.1080/22221751.2019.1643260)
Supplement: Supplemental Material [file TEMI_A_1643260_SM0218.zip › Supplementary figure legends..doc]

**Supplementary Figure Legends**

**Supplementary Fig. 1 Experimental design.**

**Supplementary Fig.2 The estrogen levels and cellular immunity in pregnant rabbits infected with HEV**

（A）Correlational analysis of HEV RNA levels of feces and estradiol. Y axis represents HEV RNA levels in feces of group A rabbits; Statistical analysis indicated that there was a certain correlation between HEV RNA levels and estradiol levels. (B) Immunohistochemistry staining of CD4^+^ and CD8^+^ positive T cells in liver, ovary and placenta tissues. Immunohistochemistry staining of CD4^+^ positive T cells in liver (a), ovary (c) and placenta (e) sections; immunohistochemistry staining of CD8^+^ positive T cells in liver (b), ovary (d) and placenta (f) sections; liver, ovary and placenta sections of negative control showed low level of staining of CD^+^4 and CD^+^8 positive T cells (g-l). Original magnification,×200.
