# Supplementary material for: Hepatitis E-related adverse pregnancy outcomes and their prevention by hepatitis E vaccine in a rabbit model
Source: Emerg Microbes Infect. 2019 Jul 24;8(1):1066–75. doi: 10.1080/22221751.2019.1643260 (PMC6711181; doi:10.1080/22221751.2019.1643260)
Supplement: Supplemental Material [file TEMI_A_1643260_SM0218.zip › Supplementary Table .doc]

Supplementary Table 1. HEV infection in surviving newborn rabbits

| Groups  (NO., HEV genotypes) | Surviving newborn rabbits (NO.) | HEV infection^a^ (%) | | | Normal newborn rabbits^b^ (%) | P value^c^ |
| --- | --- | --- | --- | --- | --- | --- |
|  |  | Shedding virus | Viremia | Anti- HEV (+) |  |  |
| A (6, rHEV3) | 4 | 2/4 (50) | 0/4(0) | 1/4(25) | 1/4(25) | - |
| B (6, sHEV4) | 5 | 0/5 (0) | 1/5(20) | 0/5(0) | 4/5(80) | 0.125 |
| C (6, hHEV3) | 7 | 0/7(0) | 0/7(0) | 0/7(0) | 7/7(100) | 0.011* |

^a^Number of newborn rabbits with HEV infection/total number of surviving newborn rabbits in the group

^b^Number of normal newborn rabbits/total number of surviving newborn rabbits in the group

^c^Compared with group A

*Significantly different, P<0.05
